# 4-(Diethyl­amino)salicylaldehyde phenyl­sulfonyl­hydrazone

**DOI:** 10.1107/S1600536808010118

**Published:** 2008-04-18

**Authors:** Xi-Shi Tai, Yi-Min Feng, Fan-Yuan Kong

**Affiliations:** a Department of Chemistry and Chemical Engineering, Weifang University, Weifang 261061, People’s Republic of China

## Abstract

In the title compound, C_17_H_21_N_3_O_3_S, the dihedral angle between the aromatic ring planes is 84.2 (2)°. The pendant ethyl groups of the –N(C_2_H_5_)_2_ group are disordered over two sets of positions in a 0.84 (2):0.16 (2) ratio. The mol­ecular conformation is stabilized by an intra­molecular O—H⋯N hydrogen bond, and inter­molecular N—H⋯O bonds lead to [010] chains in the crystal structure.

## Related literature

For related literature, see: Tai *et al.* (2008 ▶).
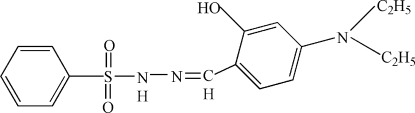


## Experimental

### 

#### Crystal data

C_17_H_21_N_3_O_3_S
*M* *_r_* = 347.43
Orthorhombic, 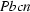

*a* = 29.874 (3) Å
*b* = 7.5153 (12) Å
*c* = 15.4456 (19) Å
*V* = 3467.8 (8) Å^3^
*Z* = 8
Mo *K*α radiation
μ = 0.21 mm^−1^
*T* = 293 (2) K
0.43 × 0.38 × 0.04 mm

#### Data collection

Bruker SMART CCD diffractometer
Absorption correction: multi-scan (*SADABS*; Bruker, 2000 ▶) *T*_min_ = 0.916, *T*_max_ = 0.992
16321 measured reflections
3052 independent reflections
2061 reflections with *I* > 2σ(*I*)
*R*_int_ = 0.048

#### Refinement

*R*[*F* ^2^ > 2σ(*F* ^2^)] = 0.069
*wR*(*F* ^2^) = 0.175
*S* = 1.08
3052 reflections
258 parameters
H-atom parameters constrained
Δρ_max_ = 0.44 e Å^−3^
Δρ_min_ = −0.30 e Å^−3^

### 

Data collection: *SMART* (Bruker, 2000 ▶); cell refinement: *SAINT* (Bruker, 2000 ▶); data reduction: *SAINT*; program(s) used to solve structure: *SHELXS97* (Sheldrick, 2008 ▶); program(s) used to refine structure: *SHELXL97* (Sheldrick, 2008 ▶); molecular graphics: *SHELXTL* (Sheldrick, 2008 ▶); software used to prepare material for publication: *SHELXTL*.

## Supplementary Material

Crystal structure: contains datablocks global, I. DOI: 10.1107/S1600536808010118/hb2720sup1.cif

Structure factors: contains datablocks I. DOI: 10.1107/S1600536808010118/hb2720Isup2.hkl

Additional supplementary materials:  crystallographic information; 3D view; checkCIF report

## Figures and Tables

**Table 1:** Hydrogen-bond geometry (Å, °)

*D*—H⋯*A*	*D*—H	H⋯*A*	*D*⋯*A*	*D*—H⋯*A*
O3—H3⋯N2	0.82	1.92	2.637 (4)	146
N1—H1⋯O1^i^	0.90	2.06	2.944 (5)	169

Symmetry code: (i) 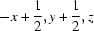.

**Fig. 1.**
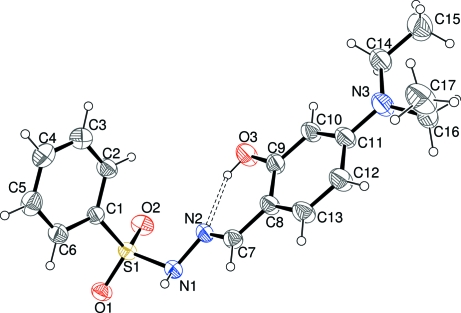
The molecular structure of (I) showing 30% displacement ellipsoids for the non-hydrogen atoms. Only the major disorder component is shown and the hydrogen bond is indicated by a double-dashed line.

## supplementary crystallographic information

### Comment

As part of our ongoing studies of aroylhydrazones as potential ligands (Tai *et al.*, 2008), we now report the synthesis and structure of the title compound, (I), (Fig. 1).

The dihedral angle between the aromatic ring planes is 84.2 (2)°. The pendant ethyl groups of the -N(C_2_H_5_)_2_ grouping are disordered over two sets of positions in a 0.84 (2):0.16 (2) ratio.  The molecular conformation is stabilised by an intramolecular O-H···N hydrogen bond and intermolecular N-H···O bonds lead to [010] chains in the crystal (Table 1).

### Experimental

3 mmol of *p*-(diethylamino)salicylaldehyde (3 mmol) was added to a solution of benzenesulfonyl hydrazide (3 mmol) in 10 ml of 95% ethanol. The mixture was continuously stirred for 4 h at refluxing temperature, evaporating some ethanol, then, upon cooling, the solid product was collected by filtration and dried *in vacuo* (yield 67%). Colourless plates of (I) were obtained by evaporation from a methanol solution after several days.

### Refinement

The H atoms were placed geometrically (C—H = 0.93–0.96 Å, N—H = 0.86 Å, O—H = 0.82Å) and refined as riding with *U*_iso_(H) = 1.2*U*_eq_(C, N) or 1.5*U*_eq_(methyl C, O).

### Crystal data

**Table:**

C_17_H_21_N_3_O_3_S	*D*_x_ = 1.331 Mg m^−^^3^
*M**_r_* = 347.43	Mo *K*α radiation λ = 0.71073 Å
Orthorhombic, *P**b**c**n*	Cell parameters from 3546 reflections
*a* = 29.874 (3) Å	θ = 2.7–21.4º
*b* = 7.5153 (12) Å	µ = 0.21 mm^−^^1^
*c* = 15.4456 (19) Å	*T* = 293 (2) K
*V* = 3467.8 (8) Å^3^	Plate, colourless
*Z* = 8	0.43 × 0.38 × 0.04 mm
*F*_000_ = 1472	

### Data collection

**Table:**

Bruker SMART CCD diffractometer	3052 independent reflections
Radiation source: fine-focus sealed tube	2061 reflections with *I* > 2σ(*I*)
Monochromator: graphite	*R*_int_ = 0.048
*T* = 293(2) K	θ_max_ = 25.0º
ω scans	θ_min_ = 2.6º
Absorption correction: multi-scan(SADABS; Bruker, 2000)	*h* = −35→34
*T*_min_ = 0.916, *T*_max_ = 0.992	*k* = −8→7
16321 measured reflections	*l* = −18→14

### Refinement

**Table:**

Refinement on *F*^2^	Secondary atom site location: difference Fourier map
Least-squares matrix: full	Hydrogen site location: inferred from neighbouring sites
*R*[*F*^2^ > 2σ(*F*^2^)] = 0.069	H-atom parameters constrained
*wR*(*F*^2^) = 0.175	*w* = 1/[σ^2^(*F*_o_^2^) + (0.0453*P*)^2^ + 7.4306*P*] where *P* = (*F*_o_^2^ + 2*F*_c_^2^)/3
*S* = 1.08	(Δ/σ)_max_ = 0.001
3052 reflections	Δρ_max_ = 0.44 e Å^−^^3^
258 parameters	Δρ_min_ = −0.30 e Å^−^^3^
Primary atom site location: structure-invariant direct methods	Extinction correction: none

### Special details

**Table:**

Geometry. All e.s.d.'s (except the e.s.d. in the dihedral angle between two l.s. planes) are estimated using the full covariance matrix. The cell e.s.d.'s are taken into account individually in the estimation of e.s.d.'s in distances, angles and torsion angles; correlations between e.s.d.'s in cell parameters are only used when they are defined by crystal symmetry. An approximate (isotropic) treatment of cell e.s.d.'s is used for estimating e.s.d.'s involving l.s. planes.
Refinement. Refinement of *F*^2^ against ALL reflections. The weighted *R*-factor *wR* and goodness of fit *S* are based on *F*^2^, conventional *R*-factors *R* are based on *F*, with *F* set to zero for negative *F*^2^. The threshold expression of *F*^2^ > σ(*F*^2^) is used only for calculating *R*-factors(gt) *etc*. and is not relevant to the choice of reflections for refinement. *R*-factors based on *F*^2^ are statistically about twice as large as those based on *F*, and *R*- factors based on ALL data will be even larger.

### Fractional atomic coordinates and isotropic or equivalent isotropic displacement parameters (Å^2^)

**Table:**

	*x*	*y*	*z*	*U*_iso_*/*U*_eq_	Occ. (<1)
N1	0.31274 (11)	0.2572 (5)	0.9493 (2)	0.0502 (9)	
H1	0.2949	0.3499	0.9363	0.060*	
N2	0.35710 (11)	0.3011 (5)	0.9253 (2)	0.0512 (9)	
N3	0.54548 (13)	0.6991 (6)	0.8710 (3)	0.0794 (14)	
O1	0.24926 (10)	0.0597 (4)	0.93219 (19)	0.0596 (8)	
O2	0.32557 (10)	−0.0581 (4)	0.9110 (2)	0.0643 (9)	
O3	0.44009 (10)	0.2276 (4)	0.8794 (2)	0.0773 (11)	
H3	0.4137	0.2060	0.8893	0.116*	
S1	0.29375 (3)	0.08000 (14)	0.89918 (7)	0.0481 (3)	
C1	0.29054 (13)	0.1295 (5)	0.7887 (3)	0.0457 (10)	
C2	0.32787 (15)	0.1092 (6)	0.7367 (3)	0.0602 (12)	
H2	0.3547	0.0683	0.7601	0.072*	
C3	0.32493 (18)	0.1505 (7)	0.6499 (3)	0.0727 (14)	
H3A	0.3498	0.1363	0.6142	0.087*	
C4	0.28604 (19)	0.2114 (7)	0.6164 (3)	0.0700 (14)	
H4	0.2846	0.2396	0.5578	0.084*	
C5	0.24886 (19)	0.2323 (7)	0.6664 (3)	0.0693 (14)	
H5	0.2224	0.2744	0.6420	0.083*	
C6	0.25055 (15)	0.1908 (6)	0.7535 (3)	0.0586 (12)	
H6	0.2253	0.2037	0.7882	0.070*	
C7	0.36859 (14)	0.4638 (6)	0.9325 (3)	0.0517 (11)	
H7	0.3473	0.5476	0.9489	0.062*	
C8	0.41362 (13)	0.5202 (6)	0.9160 (3)	0.0545 (11)	
C9	0.44774 (14)	0.4048 (6)	0.8899 (3)	0.0572 (12)	
C10	0.49056 (14)	0.4634 (6)	0.8754 (3)	0.0630 (13)	
H10	0.5124	0.3820	0.8588	0.076*	
C11	0.50227 (15)	0.6425 (6)	0.8849 (3)	0.0661 (13)	
C12	0.46791 (15)	0.7606 (7)	0.9108 (4)	0.0725 (15)	
H12	0.4742	0.8809	0.9179	0.087*	
C13	0.42560 (15)	0.6996 (6)	0.9256 (3)	0.0670 (14)	
H13	0.4038	0.7804	0.9427	0.080*	
C14	0.5780 (8)	0.571 (4)	0.8341 (15)	0.095 (6)	0.84 (2)
H14A	0.5787	0.4677	0.8715	0.114*	0.84 (2)
H14B	0.5666	0.5327	0.7784	0.114*	0.84 (2)
C15	0.6231 (3)	0.6286 (18)	0.8217 (10)	0.114 (4)	0.84 (2)
H15A	0.6242	0.7137	0.7754	0.171*	0.84 (2)
H15B	0.6414	0.5280	0.8074	0.171*	0.84 (2)
H15C	0.6339	0.6826	0.8740	0.171*	0.84 (2)
C16	0.5597 (2)	0.8789 (15)	0.8999 (8)	0.076 (3)	0.84 (2)
H16A	0.5912	0.8770	0.9147	0.092*	0.84 (2)
H16B	0.5430	0.9123	0.9513	0.092*	0.84 (2)
C17	0.5517 (3)	1.012 (2)	0.8301 (10)	0.107 (4)	0.84 (2)
H17A	0.5649	0.9712	0.7772	0.161*	0.84 (2)
H17B	0.5650	1.1239	0.8463	0.161*	0.84 (2)
H17C	0.5201	1.0281	0.8220	0.161*	0.84 (2)
C14'	0.585 (4)	0.574 (18)	0.870 (7)	0.08 (2)	0.16 (2)
H14C	0.5748	0.4534	0.8813	0.098*	0.16 (2)
H14D	0.6063	0.6079	0.9134	0.098*	0.16 (2)
C15'	0.606 (2)	0.586 (8)	0.779 (5)	0.11 (2)	0.16 (2)
H15D	0.5939	0.6879	0.7496	0.170*	0.16 (2)
H15E	0.5985	0.4801	0.7472	0.170*	0.16 (2)
H15F	0.6375	0.5966	0.7846	0.170*	0.16 (2)
C16'	0.5486 (13)	0.875 (8)	0.826 (4)	0.082 (18)	0.16 (2)
H16C	0.5207	0.9021	0.7969	0.099*	0.16 (2)
H16D	0.5724	0.8723	0.7830	0.099*	0.16 (2)
C17'	0.5588 (15)	1.015 (10)	0.896 (4)	0.089 (19)	0.16 (2)
H17D	0.5320	1.0407	0.9280	0.133*	0.16 (2)
H17E	0.5694	1.1224	0.8689	0.133*	0.16 (2)
H17F	0.5813	0.9706	0.9346	0.133*	0.16 (2)

### Atomic displacement parameters (Å^2^)

**Table:**

	*U*^11^	*U*^22^	*U*^33^	*U*^12^	*U*^13^	*U*^23^
N1	0.0406 (19)	0.055 (2)	0.055 (2)	0.0017 (16)	−0.0002 (16)	−0.0013 (18)
N2	0.0378 (18)	0.057 (2)	0.059 (2)	0.0001 (16)	−0.0014 (16)	−0.0042 (18)
N3	0.053 (2)	0.065 (3)	0.120 (4)	−0.008 (2)	0.015 (2)	−0.019 (3)
O1	0.0506 (16)	0.061 (2)	0.067 (2)	−0.0052 (15)	0.0125 (15)	−0.0017 (16)
O2	0.0634 (19)	0.0535 (19)	0.076 (2)	0.0149 (15)	0.0025 (16)	0.0112 (17)
O3	0.0547 (19)	0.053 (2)	0.125 (3)	−0.0024 (16)	0.0098 (19)	−0.022 (2)
S1	0.0427 (6)	0.0449 (6)	0.0566 (7)	0.0016 (5)	0.0036 (5)	0.0027 (5)
C1	0.044 (2)	0.038 (2)	0.054 (3)	0.0016 (18)	−0.001 (2)	−0.0076 (19)
C2	0.047 (3)	0.069 (3)	0.065 (3)	0.007 (2)	0.002 (2)	0.002 (3)
C3	0.068 (3)	0.085 (4)	0.065 (4)	−0.003 (3)	0.006 (3)	−0.001 (3)
C4	0.087 (4)	0.066 (3)	0.057 (3)	−0.001 (3)	−0.006 (3)	0.000 (3)
C5	0.074 (3)	0.063 (3)	0.071 (3)	0.017 (3)	−0.020 (3)	−0.005 (3)
C6	0.049 (2)	0.062 (3)	0.065 (3)	0.009 (2)	−0.003 (2)	−0.007 (2)
C7	0.046 (2)	0.048 (3)	0.061 (3)	0.005 (2)	0.001 (2)	−0.003 (2)
C8	0.042 (2)	0.053 (3)	0.069 (3)	0.002 (2)	0.003 (2)	−0.003 (2)
C9	0.048 (2)	0.051 (3)	0.073 (3)	0.001 (2)	−0.002 (2)	−0.011 (2)
C10	0.043 (2)	0.055 (3)	0.090 (4)	0.003 (2)	0.008 (2)	−0.018 (3)
C11	0.045 (2)	0.068 (3)	0.085 (4)	−0.003 (2)	0.008 (2)	−0.009 (3)
C12	0.056 (3)	0.053 (3)	0.109 (4)	−0.003 (2)	0.014 (3)	−0.011 (3)
C13	0.050 (3)	0.054 (3)	0.098 (4)	0.005 (2)	0.011 (3)	−0.009 (3)
C14	0.060 (9)	0.096 (8)	0.129 (17)	−0.014 (7)	0.018 (10)	−0.045 (13)
C15	0.067 (6)	0.131 (9)	0.145 (11)	0.011 (6)	−0.007 (6)	−0.040 (7)
C16	0.052 (4)	0.075 (7)	0.101 (8)	−0.006 (3)	0.001 (4)	−0.019 (5)
C17	0.072 (6)	0.097 (10)	0.152 (11)	0.004 (5)	0.011 (7)	0.011 (9)
C14'	0.06 (4)	0.08 (4)	0.11 (7)	−0.01 (3)	0.00 (4)	−0.02 (5)
C15'	0.09 (4)	0.11 (4)	0.13 (6)	−0.01 (3)	0.00 (4)	−0.03 (4)
C16'	0.06 (2)	0.08 (4)	0.11 (5)	0.00 (2)	0.01 (2)	−0.02 (3)
C17'	0.06 (2)	0.08 (4)	0.13 (5)	0.00 (2)	0.00 (3)	−0.02 (4)

### Geometric parameters (Å, °)

**Table:**

N1—N2	1.415 (4)	C10—C11	1.398 (6)
N1—S1	1.642 (4)	C10—H10	0.9300
N1—H1	0.9000	C11—C12	1.415 (6)
N2—C7	1.275 (5)	C12—C13	1.363 (6)
N3—C11	1.376 (6)	C12—H12	0.9300
N3—C14	1.48 (3)	C13—H13	0.9300
N3—C16	1.485 (11)	C14—C15	1.42 (3)
N3—C16'	1.50 (6)	C14—H14A	0.9700
N3—C14'	1.50 (15)	C14—H14B	0.9700
O1—S1	1.432 (3)	C15—H15A	0.9600
O2—S1	1.419 (3)	C15—H15B	0.9600
O3—C9	1.361 (5)	C15—H15C	0.9600
O3—H3	0.8200	C16—C17	1.49 (2)
S1—C1	1.750 (4)	C16—H16A	0.9700
C1—C2	1.382 (6)	C16—H16B	0.9700
C1—C6	1.391 (6)	C17—H17A	0.9600
C2—C3	1.380 (7)	C17—H17B	0.9600
C2—H2	0.9300	C17—H17C	0.9600
C3—C4	1.351 (7)	C14'—C15'	1.53 (16)
C3—H3A	0.9300	C14'—H14C	0.9700
C4—C5	1.362 (7)	C14'—H14D	0.9700
C4—H4	0.9300	C15'—H15D	0.9600
C5—C6	1.383 (6)	C15'—H15E	0.9600
C5—H5	0.9300	C15'—H15F	0.9600
C6—H6	0.9300	C16'—C17'	1.54 (10)
C7—C8	1.433 (6)	C16'—H16C	0.9700
C7—H7	0.9300	C16'—H16D	0.9700
C8—C9	1.398 (6)	C17'—H17D	0.9600
C8—C13	1.403 (6)	C17'—H17E	0.9600
C9—C10	1.371 (6)	C17'—H17F	0.9600
			
N2—N1—S1	112.9 (3)	C9—C10—C11	121.7 (4)
N2—N1—H1	108.5	C9—C10—H10	119.1
S1—N1—H1	108.5	C11—C10—H10	119.1
C7—N2—N1	116.9 (4)	N3—C11—C10	121.1 (4)
C11—N3—C14	118.4 (10)	N3—C11—C12	122.0 (4)
C11—N3—C16	120.2 (5)	C10—C11—C12	116.9 (4)
C14—N3—C16	121.2 (10)	C13—C12—C11	120.6 (5)
C11—N3—C16'	113.9 (16)	C13—C12—H12	119.7
C14—N3—C16'	111 (2)	C11—C12—H12	119.7
C16—N3—C16'	47 (2)	C12—C13—C8	122.8 (4)
C11—N3—C14'	123 (5)	C12—C13—H13	118.6
C14—N3—C14'	23 (4)	C8—C13—H13	118.6
C16—N3—C14'	111 (4)	C15—C14—N3	118.4 (18)
C16'—N3—C14'	120 (6)	C15—C14—H14A	107.7
C9—O3—H3	109.5	N3—C14—H14A	107.7
O2—S1—O1	119.90 (19)	C15—C14—H14B	107.7
O2—S1—N1	107.52 (19)	N3—C14—H14B	107.7
O1—S1—N1	103.83 (18)	H14A—C14—H14B	107.1
O2—S1—C1	108.52 (19)	N3—C16—C17	110.4 (12)
O1—S1—C1	108.61 (19)	N3—C16—H16A	109.6
N1—S1—C1	107.86 (19)	C17—C16—H16A	109.6
C2—C1—C6	120.2 (4)	N3—C16—H16B	109.6
C2—C1—S1	119.9 (3)	C17—C16—H16B	109.6
C6—C1—S1	119.9 (3)	H16A—C16—H16B	108.1
C3—C2—C1	119.2 (4)	N3—C14'—C15'	107 (8)
C3—C2—H2	120.4	N3—C14'—H14C	110.3
C1—C2—H2	120.4	C15'—C14'—H14C	110.3
C4—C3—C2	120.2 (5)	N3—C14'—H14D	110.3
C4—C3—H3A	119.9	C15'—C14'—H14D	110.3
C2—C3—H3A	119.9	H14C—C14'—H14D	108.5
C3—C4—C5	121.6 (5)	C14'—C15'—H15D	109.5
C3—C4—H4	119.2	C14'—C15'—H15E	109.5
C5—C4—H4	119.2	H15D—C15'—H15E	109.5
C4—C5—C6	119.7 (5)	C14'—C15'—H15F	109.5
C4—C5—H5	120.1	H15D—C15'—H15F	109.5
C6—C5—H5	120.1	H15E—C15'—H15F	109.5
C5—C6—C1	119.1 (5)	N3—C16'—C17'	107 (6)
C5—C6—H6	120.5	N3—C16'—H16C	110.4
C1—C6—H6	120.5	C17'—C16'—H16C	110.4
N2—C7—C8	121.4 (4)	N3—C16'—H16D	110.4
N2—C7—H7	119.3	C17'—C16'—H16D	110.4
C8—C7—H7	119.3	H16C—C16'—H16D	108.6
C9—C8—C13	116.2 (4)	C16'—C17'—H17D	109.5
C9—C8—C7	123.5 (4)	C16'—C17'—H17E	109.5
C13—C8—C7	120.3 (4)	H17D—C17'—H17E	109.5
O3—C9—C10	116.9 (4)	C16'—C17'—H17F	109.5
O3—C9—C8	121.3 (4)	H17D—C17'—H17F	109.5
C10—C9—C8	121.9 (4)	H17E—C17'—H17F	109.5
			
S1—N1—N2—C7	153.6 (3)	C16'—N3—C11—C10	141 (3)
N2—N1—S1—O2	52.0 (3)	C14'—N3—C11—C10	−18 (4)
N2—N1—S1—O1	−179.9 (3)	C14—N3—C11—C12	−172.9 (12)
N2—N1—S1—C1	−64.8 (3)	C16—N3—C11—C12	12.5 (9)
O2—S1—C1—C2	−31.0 (4)	C16'—N3—C11—C12	−40 (3)
O1—S1—C1—C2	−162.8 (3)	C14'—N3—C11—C12	161 (4)
N1—S1—C1—C2	85.2 (4)	C9—C10—C11—N3	179.3 (5)
O2—S1—C1—C6	149.7 (3)	C9—C10—C11—C12	0.4 (8)
O1—S1—C1—C6	17.8 (4)	N3—C11—C12—C13	−178.8 (5)
N1—S1—C1—C6	−94.1 (4)	C10—C11—C12—C13	0.1 (8)
C6—C1—C2—C3	0.0 (7)	C11—C12—C13—C8	−0.2 (9)
S1—C1—C2—C3	−179.4 (4)	C9—C8—C13—C12	−0.1 (8)
C1—C2—C3—C4	0.6 (8)	C7—C8—C13—C12	179.9 (5)
C2—C3—C4—C5	−0.6 (8)	C11—N3—C14—C15	−178.5 (15)
C3—C4—C5—C6	0.0 (8)	C16—N3—C14—C15	−4(2)
C4—C5—C6—C1	0.5 (7)	C16'—N3—C14—C15	48 (3)
C2—C1—C6—C5	−0.5 (7)	C14'—N3—C14—C15	−71 (15)
S1—C1—C6—C5	178.8 (4)	C11—N3—C16—C17	−89.7 (8)
N1—N2—C7—C8	175.4 (4)	C14—N3—C16—C17	95.8 (13)
N2—C7—C8—C9	1.0 (7)	C16'—N3—C16—C17	6(2)
N2—C7—C8—C13	−179.0 (5)	C14'—N3—C16—C17	118 (5)
C13—C8—C9—O3	179.3 (5)	C11—N3—C14'—C15'	118 (7)
C7—C8—C9—O3	−0.8 (7)	C14—N3—C14'—C15'	32 (11)
C13—C8—C9—C10	0.6 (7)	C16—N3—C14'—C15'	−90 (6)
C7—C8—C9—C10	−179.4 (5)	C16'—N3—C14'—C15'	−39 (8)
O3—C9—C10—C11	−179.5 (5)	C11—N3—C16'—C17'	101 (3)
C8—C9—C10—C11	−0.8 (8)	C14—N3—C16'—C17'	−123 (2)
C14—N3—C11—C10	8.3 (13)	C16—N3—C16'—C17'	−9(2)
C16—N3—C11—C10	−166.3 (7)	C14'—N3—C16'—C17'	−100 (4)

### Hydrogen-bond geometry (Å, °)

**Table:**

*D*—H···*A*	*D*—H	H···*A*	*D*···*A*	*D*—H···*A*
O3—H3···N2	0.82	1.92	2.637 (4)	146
N1—H1···O1^i^	0.90	2.06	2.944 (5)	169

Symmetry codes: (i) −*x*+1/2, *y*+1/2, *z*.

## References

[bb1] Bruker (2000). *SMART*, *SAINT* and *SADABS* Bruker AXS Inc., Madison, Wisconsin, USA.

[bb2] Sheldrick, G. M. (2008). *Acta Cryst.* A**64**, 112–122. 10.1107/S0108767307043930 18156677

[bb3] Tai, X.-S., Feng, Y.-M. & Kong, F.-Y. (2008). *Acta Cryst.* E**64**, o750. 10.1107/S1600536808007988 PMC2961058 21202140

